# The Origin and Early Evolution of Life: (Prebiotic) Systems Chemistry Perspective

**DOI:** 10.3390/life12050710

**Published:** 2022-05-10

**Authors:** Emiliano Altamura, Michele Fiore

**Affiliations:** 1 Chemistry Department, University of Bari Aldo Moro, Via Orabona 4, 70125 Bari, Italy; emiliano.altamura@uniba.it; 2 Institut de Chimie et Biochimie Moléculaires et Supramoléculaires, Université de Lyon, Claude Bernard Lyon 1, Batiment Lederer, Bureau 11.002, 1 Rue Victor Grignard, F-69622 Villeurbanne, France

## 1. Systems Chemistry

Aristotle considered that “nature does not do anything endless”. Today, despite the enormous scientific achievements reached in the field of the applied science, such as medicine, biology, engineering, physics and chemistry, many question marks accompany the life of scientists. Mankind has always asked one question “where do we come from? How could life have emerged from an inanimate collection of organic molecules, minerals and water?” International scientific figures, often chemists, who believe that life emerged spontaneously from a mixture of molecules in a prebiotic land agree that studies on the spontaneous origin of life are needed [1,2,3].

Molecular biology is undergoing a transformation, moving from studying cellular processes as they currently are; both basic understanding (analytical approach) and hypothesis testing by a constructive procedure (synthetic approach), using artificial models. This second route, which complements the first one, requires a degree of understanding that is ultimately based on chemistry and physics—this is why we say, “from the bottom”. For example, building artificial analogues of biochemical processes reveals whether we have really understood how a system works or whether we fail because something is still missing (according to the motto: “What I cannot create, I do not understand”, by the Nobel Prize winning physicist Richard Feynman).

“*Biology today is no more fully understood in principle than physics was a century or so ago. In both cases the guiding vision has (or had) reached its end, and in both, a new, deeper, more invigorating representation of reality is (or was) called for*”. The late biologist Carl Richard Woese (1928–2012) emphasized the urgency of conducting in-depth studies in search of what in the early days of the formation of the universe and then of our planet gave rise to what is called life [4]. In 2005, the term “**Systems Chemistry**” appeared in a conference on Prebiotic Chemistry and Early Evolution (Chembiogenesis 2005 in Venice, Italy). This field of chemical research has its roots in a number of different areas such as dynamic combinatorial chemistry, self-assembly and self-organization, prebiotic chemistry, minimal self-replicating molecules, metabolic and non-metabolic networks and autocatalytic systems. In few words, systems chemistry is the science of studying the networks of interacting molecules to form new functions from a set of molecular components at different hierarchical levels with emergent properties. The goal now is to understand, perhaps from experimental reproduction, the formation of a compartmentalized chemical system far from thermodynamic equilibrium, a system which is kinetically stable and can self-maintain (metabolism), dynamically evolve and is capable of dividing/self-reproducing, which we then could call “*alive*” or “*animate*”, according to one of the definition of life proposed in the literature in 2005 [5].

## 2. The Seminal Idea of Systems Chemistry and the Bottom-Up Approach

The main purpose of synthetic chemistry is the preparation of pure, designed compounds by using well-defined synthetic pathways and multi-step reactions. The preparation of a complex mixture of chemical compounds can be highly interesting since simple mixtures of nonreacting molecules can trigger chemical reaction networks, including feedback loops and elements of nonlinearity form. From such systems, new, unexpected and unpredicted emergent properties could arise, while none of the components alone have these properties. Systems chemistry is therefore an extremely interesting and very new way to do and to think and to rethink chemistry beyond the fundamental insight that we can obtain.

From the origin-of-life point of view, the top-down approach consists of simplifying the machinery of contemporary cells in order to obtain the minimal but efficient system which would resemble primitive cells and which could explain how they worked. Systems chemistry though can be interpreted as a **bottom-up approach**, which aims at piecing together the parts of a whole to give rise to a new and more complex system with emerging proprieties [6,7,8,9]. Concerning the study of protocellular entities, artificial lipid vesicles can be used as biomimetic systems to study and gain insights into the physiological functions of biological cells. These are cell-sized, artificial compartments consisting of natural or synthetic amphiphilic molecules, creating an artificial entity to mimic the structure and some essential properties of a natural cell, and artificial reaction networks are used to program the functions of protocells conferring specific tasks [10]. Indeed, the reduced complexity of the system allows the investigation of the dynamics of the cell behavior, minimizing the interference from the cellular complexity [11]. Since the conditions to observe the transition from inanimate to living matter are still missing, the bottom-up approach can help to clarify some aspects of this transition by building models with increasing complexity, from very early cells, not yet alive, to the first artificial living cell. Protocell systems can help us think about the minimal complexity required for the “emergence” of properties and/or the “emergence” of life. This method of proceeding tends to reproduce the process, which led to the outbreak of life and biology in chemistry.

## 3. The Aim of Prebiotic Systems Chemistry of Biomolecules

The aim of the systems chemistry of biomolecules is to combine chemistry, physics and biology to give plausible answers, supported by rigorous scientific experiments, to some questions concerning the origin of life and the evolution of synthetic chemical systems complexity. Kuhn, Pross, Pascal, Szostak and Luisi, among others, highlighted the problems of understanding the transition from chemical to biological processes and the abiotic synthesis of biomolecules. With this aim, we have asked more than fifty selected scientists to participate in this Special Issue: nineteen original articles or reviews have been published so far. We wish to thank them all for their efforts and their scientific commitment.
